# A Monolithically-Integrated μGC Chemical Sensor System

**DOI:** 10.3390/s110706517

**Published:** 2011-06-24

**Authors:** Ronald P. Manginell, Joseph M. Bauer, Matthew W. Moorman, Lawrence J. Sanchez, John M. Anderson, Joshua J. Whiting, Daniel A. Porter, Davor Copic, Komandoor E. Achyuthan

**Affiliations:** 1 Sandia National Laboratories, Integrated Microdevice Systems Department, Albuquerque, NM 87185, USA; E-Mails: mmoorma@sandia.gov (M.W.M.); jmander@sandia.gov (J.M.A.); 2 Charles Stark Draper Laboratory, Cambridge, MA 02139, USA; E-Mail: jbauer@draper.com (J.M.B.); 3 (Formerly of) Sandia National Laboratories, Albuquerque, NM 87185, USA; E-Mail: ljsanch@sandia.gov (L.J.S.); 4 3 Degrees of Separation, 444 East Second Street, Dayton, OH 45402, USA; E-Mail: joshua.whiting@3dsanalytical.com (J.J.W.); 5 Department of Mechanical Engineering, University of Louisville, Louisville, KY 40292, USA; E-Mail: daport02@louisville.edu (D.A.P.); 6 Department of Mechanical Engineering, University of Michigan, Ann Arbor, MI 48109, USA; E-Mail: copicd@umich.edu (D.C.); 7 Biosensors and Nanomaterials Department, Sandia National Laboratories, Albuquerque, NM 87185, USA; E-Mail: kachyut@sandia.gov (K.E.A.)

**Keywords:** monolithic integration, μGC, cost modeling, thermal isolation, CWA simulants

## Abstract

Gas chromatography (GC) is used for organic and inorganic gas detection with a range of applications including screening for chemical warfare agents (CWA), breath analysis for diagnostics or law enforcement purposes, and air pollutants/indoor air quality monitoring of homes and commercial buildings. A field-portable, light weight, low power, rapid response, micro-gas chromatography (μGC) system is essential for such applications. We describe the design, fabrication and packaging of μGC on monolithically-integrated Si dies, comprised of a preconcentrator (PC), μGC column, detector and coatings for each of these components. An important feature of our system is that the same mechanical micro resonator design is used for the PC and detector. We demonstrate system performance by detecting four different CWA simulants within 2 min. We present theoretical analyses for cost/power comparisons of monolithic *versus* hybrid μGC systems. We discuss thermal isolation in monolithic systems to improve overall performance. Our monolithically-integrated μGC, relative to its hybrid cousin, will afford equal or slightly lower cost, a footprint that is 1/2 to 1/3 the size and an improved resolution of 4 to 25%.

## 1. Introduction

Gas chromatography (GC) is a widely employed technique with a variety of civilian and defense related applications. These include indoor/outdoor surveillance, patient breath analysis for volatile organic compound (VOC) diagnostics, chemical warfare agents (CWA) detection, and home/commercial buildings air quality monitoring [1–3]. Briefly, GCs separate air or gas samples and utilize a pre-concentrator (PC) to concentrate the analytes to improve detection in a high flow rate environment [4]. The concentrated sample is then flash desorbed by rapid heating of the PC. The sample plug passes through a GC column coated with a wall coating suitable for separation of the analytes. To aid in the separation of analytes with a wide range of boiling points, the temperature of the GC column is raised in a monotonic manner. The lower temperatures at the beginning of the temperature ramp allow more volatile compounds to be released from the column coating more quickly, enhancing their separation, while higher temperatures at later times increase the rate at which less volatile compounds travel through the column. This facilitates both higher and lower boiling point analytes to be detected in a single analysis and over a shorter period of time relative to isothermal analysis. The separated analytes pass over a sensor/detector array that permits identification of the sample composition. Finally, the data analysis device translates the signals into a GC chromatogram for identification of the analytes and archival purposes.

Conventional GCs are large, benchtop, expensive, heavy, high power, and fragile instruments, ill suited for field deployment for onsite gas monitoring. In CWA detection, it is essential to have a micro-gas chromatography (μGC) system that avoids these limitations and be capable of use by First Responders. Additionally, μGC systems find civilian uses for environmental monitoring in homes, office buildings or factories in a stand alone mode of field settings. Toward this goal, Terry and Herman [5] first reported a μGC fabricated on a Si wafer. Since then, we and others have been developing μGC systems possessing the attributes of rapid response, small footprint, low cost, low power, light weight, battery-operated units [3,6–12]. These μGC systems also possess higher resolution with narrow, small columns that have low dead volume and improved sensitivity with smaller sample volumes. However, most μGCs are hybrid systems and fabricate separately the PC, the column and the detector from different types of materials and subsequent assembly of the subsystems. To illustrate, the entire μGC system was assembled from separately fabricated subsystems [9] or the PC alone [10] or the PC and the column were fabricated together [11] or the column alone was micromachined [12], with some GCs weighing 10 kg [13].

To overcome these limitations we now describe the design, fabrication and monolithic integration of the PC, GC column and detector using Si dies. Monolithic integration in Si permits further miniaturization of the system, cost reduction in fabrication and assembly with simpler, smaller fluidic connections and reduced dead volumes [5,14]. We also describe the packaging of the monolithic integrated system and document its performance by separating and detecting four different CWA simulants. We present a cost analysis model, discuss opportunities and challenges confronting monolithic integration and comment on ideas to overcome thermal isolation arising from monolithic integration.

## 2. Experimental Section

### 2.1. Device Fabrication

**Figure 1.**
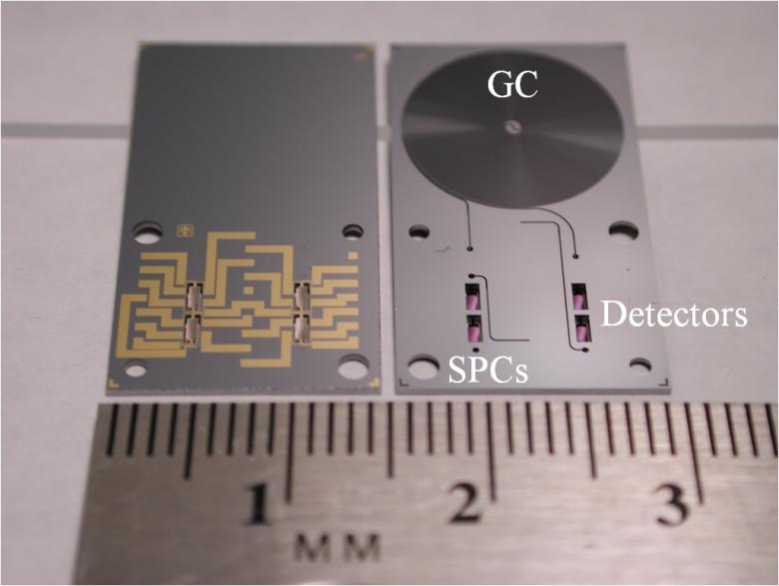
Two monolithically-integrated μGCs chips side-by-side. The chip on the left-hand side has the metallic traces used to electrically connect the PC and sensor shown face up. The chip on the right hand side is metal-side down to show the fluidic channels and deep reactive ion etched (DRIE) μGC channel.

Our monolithically-integrated system in Si is shown in Figure 1. The GC column is 100 μm wide by 400 μm deep in cross section and 86 cm long. The detector is a magnetically-actuated pivot plate resonator (PPR) [7,15] and relies on Lorentz forces generated by an alternating current in the presence of a magnetic field to drive the detector platform into mechanical oscillation. A polymer coating (described below) on the detector temporarily traps the analytes and the attendant mass change induces a shift in oscillation frequency, which is detected electronically. The PPR detectors have sensitivity similar to the surface acoustic wave (SAW) detectors that are fabricated from piezoelectric materials (common in hybrid GCs) [16,17]. An important feature of our system is that the PPRs can be used as mass-sensitive GC detectors, and, by adding a thin-film desorption heater to a PPR, it can also be turned into a mass-sensitive preconcentrator [7]. Using the same MEMS PPR platform for both the preconcentrator and detector greatly simplifies fabrication.

The fabrication steps required to produce this system are as follows:

The monolithic system is fabricated in a silicon-on-insulator (SOI) wafer, 500 μm thick, with a 5 μm device layer and 1 μm buried oxide (BOX) layer. Systems with and without an electrically-insulating layer (situated between the metal transducer lines and underlying Si) were produced. Adding such a layer increases the processing steps, so this scheme will be described in detail. The wafers are coated on both sides with a Si-rich, silicon nitride layer deposited by low pressure chemical vapor deposition (Delft University of Technology, The Netherlands). The low stress characteristic of this film minimizes undesired changes in resonance frequency of the PPR due to film-induced strain and provides electrical isolation from the device layer. The thickness of the Si_3_N_4_ available was 500 nm, but could be made much thinner.

After standard semiconductor cleaning, the transducer and heater lines are produced by liftoff of a Cr/Au, e-beam evaporated metal. Typical thicknesses of the Cr adhesion layer and Au conductor layer are 15 nm and 750 nm, respectively. This thickness provides the high conductance required for the transducers and eliminates the need for a secondary bond pad layer. Following liftoff and cleaning, the boundaries of the PPR and circular through-wafer holes are defined in photoresist and the silicon nitride layer is etched by reactive ion etching (PlasmaTherm 790) to reveal the underlying Si device layer. The photoresist is reapplied and the Si device layer is etched using a Bosch Deep Reactive Ion Etcher (PlasmaTherm Bosch or Alacatel AMS 100). Etching is concluded when the underlying BOX is revealed by the DRIE. At this point, the PPR paddle is defined and is supported on the BOX layer and by the two Si tethers to the substrate. Circular holes in the device layer are also etched in locations that will subsequently become through vias after matching locations are etched from the backside of the wafer. The penultimate etching step is a blanket strip of the silicon nitride from the reverse side of the wafer, exposing the Si wafer and permitting subsequent anodic bonding of Pyrex. Using a Karl Suss MA/6 back-side aligner, rectangular patterns are defined in photoresist and centered on the PPRs while circular holes align to the through-wafer holes; the GC column is also defined in this step. Through-wafer DRIE terminates on the BOX layer leaving the PPR suspended by its tethers and the remaining BOX, leaving a thin-layer of BOX between the two sides of the circular through holes. To free the PPRs and allow through-wafer holes, the remaining BOX is removed with either RIE or buffered oxide etching. The smaller size of the GC column (compared to the through wafer holes) results in a slower etch rate of the column with a depth of 400 μm.

To provide the fourth wall of the μGC column and system access ports, two final processing steps are performed. First, Pyrex 7740 wafers with ports aligning to the etched SOI wafer are created, typically by ultrasonic machining (Bullen Ultrasonics, Eaton, OH, USA). Second, the SOI wafer and the Pyrex are anodically bonded. A microwave downstream etcher, configured with N_2_O, is used to generate an oxygen plasma to clean the bonded surfaces. A Piranha solution (2:1, H_2_SO_4_ to H_2_O_2_) is heated to 130 °C and the wafers are inserted for 10 min. Following deionized (DI) water rinse and dry, 64:4:1 H_2_O:H_2_O_2_: NH_4_OH, heated to 45 °C on hotplate for 10 min is used. A final DI rinse and dry step is performed. The wafers were bonded in an Electronic Visions Bond/Aligner under vacuum at 350 °C for 15 min. Dicing on a standard dicing saw is used to separate the individual systems from the wafer.

### 2.2. Packaging

**Figure 2.**
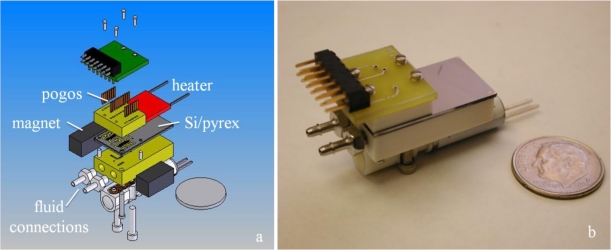
(**a**) Expanded solid model showing the Si/pyrex device, electrical and fluidic connections, external valve for switching between PC and detector modes, magnets for PPR actuation, and a disk the size of a US dime to illustrate size. (**b**) Device and packaging. Magnets and heater are not shown.

The packaging of the μGC is shown in Figure 2. The device is held between two pieces of machined material Techtron (polyphenylene sulphide) or PEEK (polyether ether ketone). Deep-etched through holes in the device accept pins in the packaging to control the alignment between the device and the packaging. Electrical connections are made on the device side and fluidic connections on the column side of the chip. “Pogo pins” (AlphaTest Corporation Mesa, AZ, USA) electrically connect the metal traces on the device with pads on the printed circuit board (PCB) (ExpressPCB, Santa Barbara, CA, USA). The connection to external electronics is then completed with a ribbon cable that plugs into a right angle header on the edge of the PCB board. Fluid flow in and out of the system is through threaded barbed fittings (Beswick Engineering Greenland, NH, USA) that connect to channels drilled and/or milled into the packaging. Lastly, an external valve (Lee Company, Westbrook, CT, USA) permits switching between collection/PC and detector modes of the system. A resistive heater (MINCO Products, Minneapolis, MN) is adhered to the GC side of the chip to heat the column during system operation. We intend to replace the external heater with metal traces patterned directly on the Si.

### 2.3. Coating Fixtures

**Figure 3.**
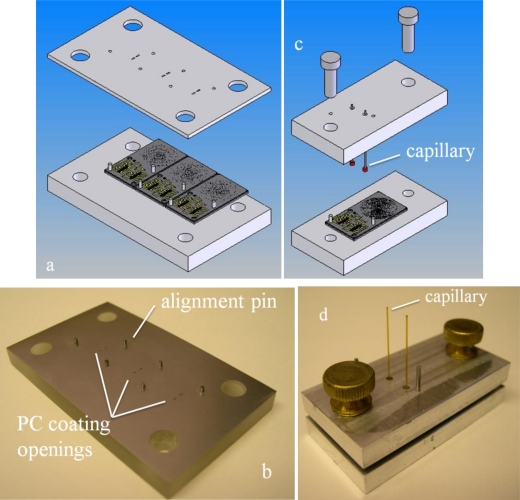
Solid models and actual images of the coating fixtures. The PC coatings were applied with a spray coating system and a shadow mask as illustrated in (**a**), solid model, and (**b**), photograph of the machined coating assembly. As shown in (a), multiple devices can be coated simultaneously. Coating of the GC column is accomplished with temporary fluidic connections (**c**) and (**d**). Two short sections of polyimide-coated, fused silica capillary can be seen protruding from the coating fixture lid. These are used to temporarily connect the GC to the sources of stationary phase coating materials.

The PC, GC column, and PPR/detector subsystems have unique polymer coating applied to their surfaces to enable the functionalities described above. The PC/detector PPRs were both coated with silica sol gel adsorbent materials [7] by means of a spray coating system and shadow masks. The shadow masks have small laser-cut windows that align with the PPRs and permit selective coating of the areas of interest. Multiple devices can be coated simultaneously with such a configuration [Figure 3(a,b)]. The GC column is coated with a stationary phase, described shortly, in a fixture that allows a temporary fluidic connection to be made to the device [Figure 3(c,d)]. From the standpoint of fixture and device design, the polymer coatings can be applied relatively easily with these designs provided adequate spacing exists between the various subsystems. Of more serious concern is the coating yield (the number of devices that are successfully coated). The columns were first filled with a solution of dimethyl(dimethylamino)vinyl silane free of air bubbles and heated at 60 °C for 1 h and then purged with He at 50 psig for 30 min. Then the columns were coated dynamically with a solution of polydimethylsiloxane (PDMS) mixed with methylene chloride and pentane (0.01:3.94:3.94) containing 1.6% (v/v) of azodiisobutyronitrile (AIBN) under 30 psi nitrogen head pressure and free of air bubbles. The column is then heated to 40 °C under vacuum for 2.5 h and later baked at 120 °C for 10 min.

## 3. Results and Discussion

### 3.1. Chemical Warfare Simulants Analysis Using Monolithically Integrated μGC

**Figure 4.**
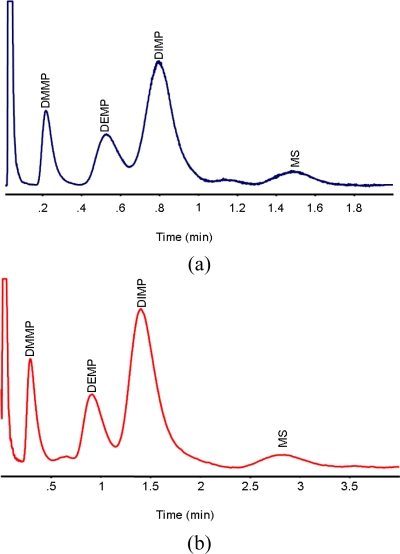
(**a**) Separation of four different CWA simulants dimethyl methyl phosphonate (DMMP), diethyl methyl phosphonate (DEMP), diisopropyl methyl phosphonate (DIMP) and methyl salicylate (MS) under 40 psi head pressure and a temperature ramp of 100 °C/min. The carrier gas was H2. (**b**) Separation of the same four different CWA simulants in under 20psi head pressure and temperature ramp of 10 °C/min. The carrier gas was H_2_.

In order to understand the performance of the PC and μGC, their output was connected to a standard, commercial flame ionization detector (FID). Figure 4(a,b) demonstrate the separation of four critical CWA surrogates on the monolithic μGC under two different separation conditions. The data of Figure 4(a) demonstrates adequate separation of four different CWA stimulants, identified in the figure caption, in only 1.5 min using the monolithic μGC system. A typical sample used for this analysis consisted of approximately 2 μL of each analyte dissolved in 2 mL of carbon disulfide. Typically 0.2 μL of this solution would be injected into the system with a split ratio of about 20–500:1. In this case of DMMP, this equates to 1–20 ng of mass on the column. Section 3.2, below, describes the use of a Golay plot for determining optimum GC operation conditions. The data of Figure 4(b) was taken under the conditions of the minimum of the Golay plot. The chromatography is completed in just under three minutes and is noticeably improved. It should be emphasized that the temperature ramp for Figure 4(b) was also ten times slower than Figure 4(a), impacting the separation time and performance.

### 3.2. μGC Efficiency

**Figure 5.**
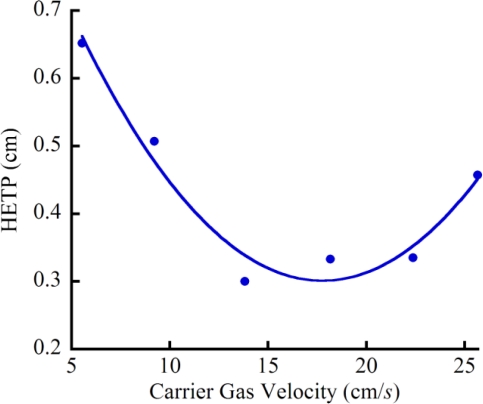
Golay plot of the relationship between the separation efficiency of the monolithically integrated GC column and the carrier gas flow rate.

The separation efficiency (*i.e.*, height equivalent to one theoretical plate, HETP) of a GC column is controlled by three main parameters: column length, column internal diameter and carrier gas flow rate. Smaller internal diameter columns generally provide higher resolution. The relationship between separation efficiency, internal diameter, and carrier gas rate is shown in the Golay plot with a good separation performance at the minimum despite a slight leak in the fixturing (Figure 5).

### 3.3. Comparing Monolithically Integrated and Hybrid Integrated μGC Systems

**Figure 6.**
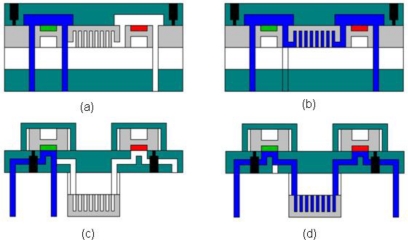
Side view schematics of monolithically-integrated (**a**) & (**b**) and hybrid integrated (**c**) and (**d**) assembling and packaging of μGC systems. Images (a) and (c) show the systems in sample mode (with gas flow (colored blue) past the PCs (“green” adsorbent) and exiting into the waste stream), while (b) and (d) show analysis modes with flow through the μGC column (illustrated with “fin-like” structure) and past the sensors (“red” adsorbent).

There are several advantages to hybrid systems including modular replacement of various components and thermal isolation of individual components operating under different temperatures. However, monolithic integration in Si permits miniaturization, batch fabrication, fewer assembly steps, cost reduction in materials and labor, smaller fluidic components, significantly reduced system dead volume, better leak tolerance, while simultaneously linking PC, GC and detector thermally through conductive heat transfer. The temperature of the detector, particularly asorption-based detector such as the PPR described in this paper, can be influenced by temperature ramping of its monolithic GC counterpart, if care is not taken in thermal isolation. With temperature cycling of the GC, one can assure that the PC adsorbent is cool during the collection phase, so heat transfer between GC and PC is not as much of an issue as with a sorptive detector. With the current μGC system, our effort has progressed to the point where comparisons (see Figure 6) can be made between monolithically-integration and the more mature hybrid μGC systems [7,15].

### 3.4. Thermal Isolation in Monolithically Integrated μGC

**Figure 7.**
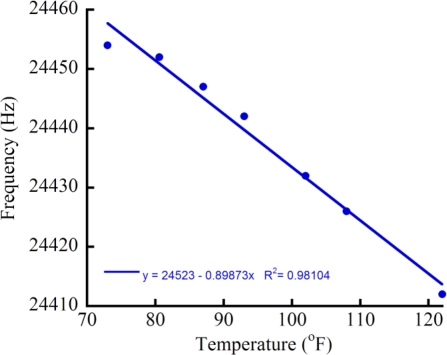
Effect of temperature on the resonant frequency of the sensor. When the monolithically-integrated μGC column is heated, conductive heat transfer through the silicon heats the sensors, shifting the resonance frequency.

Due to the high thermal conductivity of Si, heating of the μGC column will rapidly transfer heat to the PPR side of the device through conductive heat transfer. This is detrimental to system performance because of two separate effects of increasing the temperature. First, the resonant frequency of the PPR detector shifts with increasing temperature. Since a shift in frequency signals the passage of analyte, the temperature ramp of the μGC column will produce a shifting baseline of the output signal. While this shifting baseline can be (at least partially) canceled electronically through the use of a reference detector, it is not a perfect solution due to the second effect of decreased trapping efficiency leading to decreased to signal-to-noise (S/N), thus making the detection of low concentrations of analytes difficult (Figure 7). Consequently, the various subsystems must be decoupled thermally for optimal system operation.

**Figure 8.**
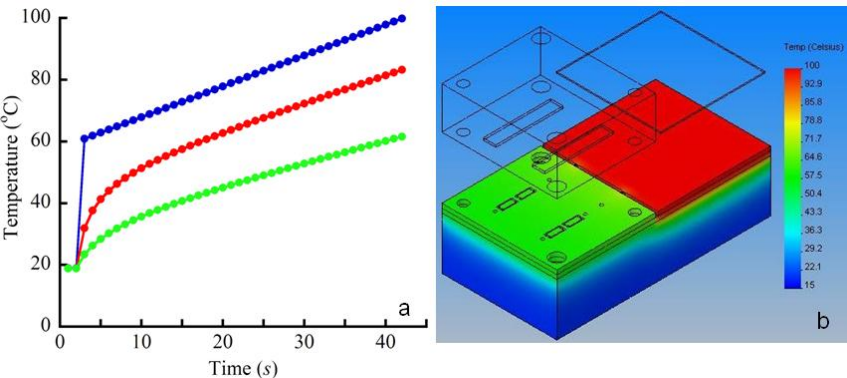
Temperature of μGC column compared to detector temperature for two thermal isolation schemes (**a**) and CosmosWorks color image of heat gradients (**b**). The blue curve represents the GC temperature ramp and red and green tracings are the detector temperature with a 400 × 100 μ thermal isolation cut and 500 × 300 μ cut, respectively. Both schemes involve physical cuts in Si between the column side and the PC/detector side of the chip. The most effective thermal isolation is to cut trenches through the entire thickness of Si except in small regions to permit fluidic coupling between the two sides of the chip. Physical barriers to thermal conduction and heat sinks on the PC/detector side of the chip permit the subsystems operating at desired temperatures with minimal increase in complexity/cost.

There are a number of thermal isolation schemes that can be implemented in Si devices. Similar isolation schemes were implemented in Si chip-based polymerase chain reaction (PCR) for DNA analyses [18,19]. The simplest thermal isolation scheme involves cutting physical breaks in Si between the various subsystems to limit the rate of conductive heat transfer. Trenches in Si can be cut during the same processing step as the GC column and through holes with DRIE. The effects of two types of trenches on conductive heat transfer in our μGC were modeled with FEA software (CosmosWorks, Concord, MA, USA, Figure 8). The trenches were placed between the μGC and PPR side of the device, with the exception of small areas to permit fluidic channels between the subsystems. One trench configuration was the same as the GC channel (100 μm wide, 400 μm deep), while the other was 400 μm wide and cut through the entire thickness of the Si wafer. The 400 μm width was chosen as the minimal dimension for through holes in Si due to the faster etching of larger features in the DRIE process.

Conductive heat transfer through the Si/pyrex anodically bonded device as well as through the Techtron fluidic blocks in contact with the device were modeled as the temperature of the GC heater was ramped from 60 °C to 100 °C over the course of 40 seconds. This temperature ramp is commonly used in the hybrid systems [7,15]. The values of thermal conductivity (K (W m^−1^ C^−1^)) for Si, Pyrex, Techtron and Al are 146, 1.01, 0.30 and 236, respectively. The boundary conditions for all surfaces exposed to air were described by heat transfer due to natural convection: −k × ∇*T* = *h*(*T* − *T*_ambient_) with a convective heat transfer coefficient, *h*, of 30 Wm^−2^·K^−1^, which is a typical value for miniature systems [20] and a *T_ambient_* of 18 °C. Given the size of the PPR chambers, only conductive heat transfer was simulated in these regions. As expected, the smaller cross sectional area available for conductive heat transfer with the 400 μm wide trenches reduced the final temperature on the PPR side of the device more than the 100 μm wide, 400 μm deep trenches (Figure 8).

The 40 degree rise in temperature on the PPR side of the device with the larger trenches can be further reduced through the use of convective heat transfer with heatsinks. The vast majority of heatsinks used to cool the Si chips in personal computers are Al (some painted or colored to enhance heat loss due to radiation). This is because Al has a high thermal conductivity (*vide supra*), is lightweight, relatively inexpensive, and relatively easy to machine/form. Further reductions in the temperature on the PPR side of the device can be accomplished effectively and inexpensively by adding Al heatsinks to the packaging. This is an attractive option as the packaging is simpler and cheaper to modify than the Si device.

To heat the GC column according to the temperature ramp shown in Figure 8 requires about 22 Joules of energy. Over the course of 40 s, this translates into approximately 0.54 Watts of power on average to dissipate. Heat dissipation through convective heat transfer is given by:

Q=hAΔT

where *Q* is the convective heat loss, *h* is the convective heat transfer coefficient, and *A* is area. With the calculated final temperature (60 °C) on the PPR side from the FEA analysis, the horizontal area required is 428 mm^2^ for an *h* of 30 Wm^−2^·K^−1^. An area of 428 mm^2^ is approximately 18% larger than the area of the Si chip. With the vertical fins of a heatsink, more area for convective heat transfer is available in a given footprint, but the effective heat transfer coefficient (and heat dissipation) is lowered due to the stagnant boundary layers that form close to the vertical fins as the more buoyant air heated by the fin surface rises away from the heatsink [21]. With forced convection, *i.e.*, forced fluid flow past the fins, the heat transfer rate will increase. Forced convection would however require the addition of a fan to push air past the fins of the heatsink. A fan does not increase the power required for a monolithic design to an amount greater than that required for a hybrid system. In the hybrid integrated system, the capillary interconnects between the subsystems are heated to avoid cold spots and condensation. Furthermore in the hybrid system, the SAW detectors are heated to ∼40 °C to avoid variations in detector temperature caused by varying ambient temperatures. If necessary, the PPR detectors could operate in a similar manner, but with less power required for heating. PPR detectors could be heated to a temperature greater than ambient through resistive heating of thin metal traces on the detector. The traces would only heat the small mass of the plate instead of the thick surrounding bulk substrate.

### 3.4. Cost Comparisons of Hybrid *versus* Monolithically Integrated μGC

One important consideration with monolithic integration involves the yield, or percentage of functional devices, after fabrication. For example, if a PC is nonfunctional on a monolithically-integrated device, the entire μGC (PC, GC, and PPR) must be discarded. On the other hand, with a hybrid integrated approach, each of the subsystems can be fabricated separately. On a wafer full of identical PCs, the fraction of PCs that are nonfunctional are simply discarded. This relationship can be thought of in terms of a true cost per part as described by Equation (1):

(1) $$C_{t}=C_{p}\cdot \left(\frac{1}{Y_{f}}\right)\cdot (\frac{2}{Y_{c}}).$$

For a given fabrication per part cost (*C_p_*), the true cost of the part (*C_t_*) is increased by the losses associated with fabrication (*Y_f_*) and coating yields (*Y_c_*). The true cost per part for monolithic-integration is increased due to the fact that a product of the individual yields is required, as shown in Equations (2) and (3):

(2) $$Y_{f}(m)=Y_{f}(m,PC)\cdot Y_{f}(m,GC)\cdot Y_{f}(m,PPR)$$

(3) $$Y_{c}(m)=Y_{c}(m,PC)\cdot Y_{c}(m,GC)\cdot Y_{c}(m,PPR)$$

The *m* in Equations (2) and (3) refers to monolithically-integrated systems. The resulting true sensor cost for the monolithically-integrated system is then calculated with Equation (4):

(4) $$C_{t}(m)=C_{p}(m)\cdot \left(\frac{1}{Y_{f}(m)}\right)\cdot \left(\frac{1}{Y_{c}\left(m\right)}\right)$$

For the hybrid, the true sensor cost is given by the sum of the individual true costs per part:

(5) $$C_{t}(h)=\frac{C_{p}(h,PC)}{Y_{f}(h,PC)\cdot Y_{c}(h,PC)}+\frac{C_{p}(h,GC)}{Y_{f}(h,GC)\cdot Y_{c}(h,GC)}+\frac{C_{p}(h,SAW)}{Y_{f}(h,SAW)\cdot Y_{c}(h,SAW)},$$

where *h* refers to the hybrid system. The relationships described by Equations (1) through (5) are relevant only to the fabrication costs of the micromachined parts. The packaging of these parts is a significant contribution to the overall system cost. To the contrary, the packaging cost of monolithic integration is a fraction of the hybrid system cost since only one micromachined part has to be packaged, compared to the three separate parts of the hybrid system. In addition, the labor costs of packaging three parts are higher than the costs associated with packaging only one part.

**Figure 9.**
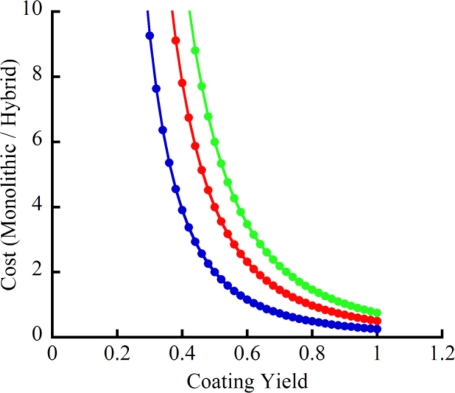
Effect of coating yield on the relative cost of monolithic *versus* hybrid integrated systems. The graph is simply a plot of a/x3, where x is the coating yield (assumed for simplicity to be the same for all three coating steps) and a is the ratio in part cost between the monolithic chip and the three hybrid chips. The general trends shown will hold true as the analysis is extended to include the cost of the individual coating steps, the packaging, and more realistic ratios in part cost. The blue, red and green tracings represent a values of 0.25, 0.5 and 0.75, respectively.

It is useful to consider the relative costs of the two approaches. A relative cost is a more meaningful comparison than actual costs, since the price of producing parts in low volume will be significantly higher than the costs of eventual mass production. A relative cost comparison assumes that future increases in yield and decreases in dollar costs will be similar for the two approaches. This is a reasonable assumption since similar processes and materials are used in both systems. The current cost of the monolithic approach (μGC sensor and packaging) is ∼200% of the hybrid cost. However, this is due to the fact that the monolithic integration has not been in development for as long as the hybrid system. With improvements in fabrication and coating yields, the cost of the monolithic system could drop to ∼80% of the cost of the hybrid system. To illustrate this effect, Figure 9 shows how improvements in coating yield impacts the system cost. The graph is a plot of *a*/*x*^3^, where *x* is the coating yield (assumed for simplicity to be the same for all three steps) and *a* is the ratio in part cost between the monolithic chip and the three hybrid chips. The general trends shown in Figure 9 will hold true as the analysis is extended to include the cost of the individual coating steps, the packaging, and more realistic ratios in part cost.

A fabrication/cost consideration that is more difficult to quantify involves the size of the system. The monolithic version is ∼1/3 the size of the current hybrid systems. However, the handheld unit that the μGC interfaces with is considerably larger than either the monolithic or the hybrid system. This handheld unit is about the size of a graphing calculator and contains other components necessary for μGC operation, such as a fluidic pump, particulate filters, control electronics, batteries, a user interface, *etc*. With the current handheld unit, the size reduction achieved with monolithic integration does not significantly reduce the size of the overall unit. Further reductions in the size of the handheld unit, would make the smaller size of the monolithic system more significant.

### 3.4. Performance Enhancements with Monolithically Integrated μGC

Ideally, the various analytes pass through the detector as narrow concentrated bands that are completely separated from adjacent bands exiting the column. This ideal scenario would exhibit maximum S/N and provide a high degree of analytes’ resolution. Under real world conditions however, diffusion and the rates of mass transfer in the stationary and gas phases contribute to “band broadening.” Faster rates of mass transfer between the stationary and the gas phases and lower rates of diffusion keep the individual analyte molecules closer together and minimize band broadening. The effects of band broadening and the efficiency of a GC column can be described by the Golay Equation [22] for open rectangular cross section GC columns as follows:

(8) $$H=\frac{Bf_{1}f_{2}}{u}+\left(\frac{C_{g}f_{1}}{f_{2}}+C_{s}\right)u.$$

*B* in Equation (8) describes the molecular diffusion of the analytes while *C* refers to the rates of mass transfer in the stationary (*C_s_*) and gas phases (*C_g_*) phases. The average linear gas velocity is *u*, and the *f_1_* and *f_2_* are gas compression correction terms that account for increases in the diffusion coefficient as the gas pressure decreases along the length of the column. Smaller values of *H* produce more efficient column performance with less band broadening. Band broadening can also occur in the fluidic interconnects between the subsystems as well as in the fluidic chambers surrounding the detectors. This band broadening is accounted for through the addition of another term [23] to Equation (8) as shown below:

(9) $$H=\frac{Bf_{1}f_{2}}{u}+\left(\frac{C_{m}f_{1}}{f_{2}}+C_{s}\right)u+\frac{\Delta t^{2}u^{2}}{L{\left(k+1\right)}^{2}}.$$

This additional term relates to the dead time (Δ*t*) associated with sample transport through the fluidic channel between the GC and the detector and the fluidic chamber of the detector. A monolithically-integrated system has a smaller Δ*t* due to its smaller fluidic interconnects and detector chamber volumes.

Another measure of GC column efficiency is its resolution, R_s_. Resolution refers to the degree with which adjacent measurements are separated; *i.e.*, the degree to which adjacent analyte bands are separated. The *Rs* is related to *H* as shown in Equation (10):

(10) $$R_{s}\propto \frac{1}{\sqrt{H}}$$

From Equations (8) and (9), improvements to resolution in monolithic integration relative to the hybrid design can be calculated assuming the values of *B* and *C* of the two systems are identical. This is a reasonable assumption since identical GC columns are used in both. Current hybrid integrated systems have a plate number of ∼1,300–4,500 per meter [24]; the theoretical maximum plate number for the current channel geometry is 8,000 plates per meter. With our monolithic integration, the time to sweep the interconnect and the chamber volume is 41% less than the time required in the hybrid system. This smaller Δ*t* yields a 4% increase in resolution with the current plate height and a 25% improvement in *Rs* as the plate number approaches the theoretical maximum.

## 4. Conclusions

A monolithically-integrated μGC can be made with an equivalent or slightly reduced cost, a physical footprint of approximately one-half to one-third the size of a hybrid integrated system and an improved resolution of 4 to 25% relative to the hybrid. The fabrication, coating, and packaging processes have matured to the point that the focus can be shifted to address performance aspects of the GC analysis. Future improvements to the current system will include the addition of thermal isolation as well as further reductions in dead volume to further increase the efficiency of the system. Volume fabrication of the monolithic integration will drive down the cost of the overall μGC production.
